# Bioconversion of Flavonoid Glycosides from *Hippophae rhamnoides* Leaves into Flavonoid Aglycones by *Eurotium amstelodami*

**DOI:** 10.3390/microorganisms7050122

**Published:** 2019-05-05

**Authors:** Qiuya Gu, Guoliang Duan, Xiaobin Yu

**Affiliations:** The Key Laboratory of Industrial Biotechnology, Ministry of Education, School of Biotechnology, Jiangnan University, Wuxi, Jiangsu 214122, China; glduan123@163.com

**Keywords:** *Eurotium amstelodami*, *Hippophae rhamnoides* leaves, bioconversion, flavonoid glycosides, flavonoid aglycones, antioxidative activity

## Abstract

The flowering process has been reported to play crucial roles in improving the flavor and efficacy of fermented tea. *Hippophae rhamnoides* leaves containing many beneficial ingredients are a suitable plant source for tea processing. In this study, we isolated a β-glucosidase-producing fungus *Eurotium amstelodami* BSX001 from the fermented tea and used *Hippophae rhamnoides* leaves (HRL) as a substrate to explore the detailed process of bioconversion of some important functional factors. The results show that the contents of total phenolic compounds and flavonoids increased significantly after seven days, especially flavonoid aglycones (e.g., quercetin, kaempferol, and isorhamnetin). Such compounds greatly enhance the antioxidative activity of fermented products. Metabolic analysis of the standard compounds (rutin, quercetin-3-glucoside, kaempferol-3-glucoside, quercetin, isorhamnetin-3-glucoside, isorhamnetin, and kaempferol) further confirmed the effective biotransformation by *E. amstelodami*. Mechanisms of the bioconversion could be involved in deglycosylation, dihydroxylation, and O-methylation. Our findings expand the understanding of tea fermentation process and provide further guidance for the fermented tea industry.

## 1. Introduction

**Figure 1:**
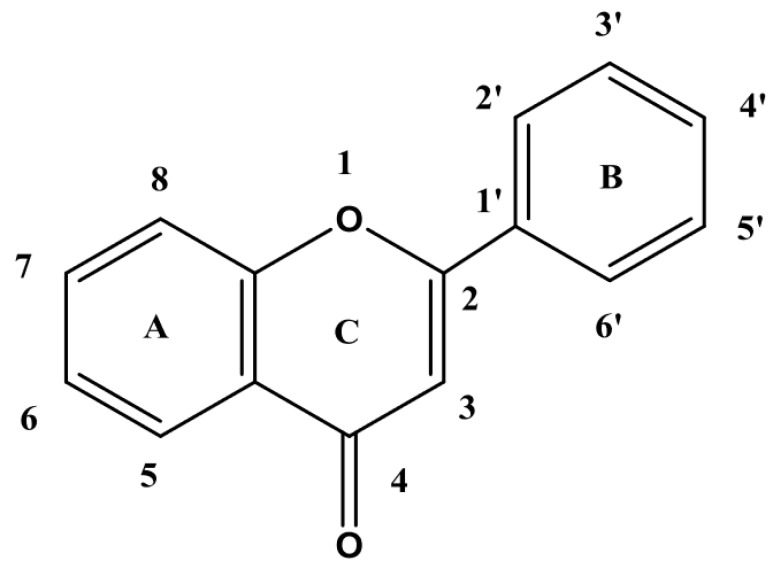
Nuclear structure of flavonoids [3].

Flavonoids are phenolic compounds that are widely found in nature. They are secondary plant phenolic compounds widely distributed in plant leaves, seeds, barks, and flowers. Over 4000 kinds of flavonoids have been determined by now. These compounds have been found to protect plants from the radiation of ultraviolet from sunlight and the damage of pathogens or herbivores [1,2]. In structure, flavonoids are benzo-γ-pyrone derivatives composed of both phenolic and benzene rings (Figure 1) [3]. Dietary flavonoids vary in the positions of hydroxyl, methoxy, and glycosidic groups and also differ in the conjugation between the A- and B-rings. During metabolism, hydroxyl groups are added, methylated, sulfated or glucuronidated [4]. Based on their unique chemical structures, flavonoids show a variety of catalytic properties for scavenging reactive oxygen and nitrogen species. The presence of an ortho-hydroxylation on the B-ring of flavonoid molecule, a C2-C3 double bond on the C-ring, the number of free hydroxyl groups or the presence of a 3-hydroxyl group is usually essential for antioxidant and antiradical activities [5,6]. Due to these numerous properties, flavonoids have been found to be emerging natural compounds in a wide variety of industries.

*Hippophae rhamnoides* L., which is also known as sea buckthorn, is an ancient plant with modern virtues in nutrition and medicine. All parts of this wonder plant are considered to be a good source of a large number of bioactive compounds, such as carotenoids, tocopherols, sterols, flavonoids, lipids, vitamins, tannins, and minerals, which contribute to its wide usage as a natural antioxidant [4,7]. The leaves of *H. rhamnoides* (HRL) have remarkable contents of phenolic compounds. Phenolic compounds in HRL have shown significant antioxidant properties and can be used to prevent the damaging effect of oxidant radicals [8,9]. Under the optimum extraction condition (74.2 ℃ for 30 min), the values of the antioxidant potential and the total phenolic compounds were 85.34% and 72.13 mg/g gallic acid equivalent (GAE), respectively. The antioxidant potential of aqueous extract of HRL varies from 76.44% to 88.82% of the potential of original compounds, with the total content of phenolic compounds ranging from 67.91 to 88.69 mg GAE/g [10]. The chemical composition of subcritical water extracts (SWE) showed a total content of phenolic compounds (76.07–93.72 mg/g GAE) and flavonoids (47.06–66.03 mg/g rutin) [7]. The content of rutin in HRL is up to 1.64 mg/g. The total content of flavonoids in HRL varies from 8 to 13.8 mg/g, which is determined by several factors, such as the geographical location, harvesting time, and light. In content, quercetin is the most abundant flavonol, followed by isorhamnetin, and kaempferol. These compounds generally occur in the glycosylated form, bound to glucose, rhamnose, or rutinose [11].

It has already been demonstrated that flavonoids have a wide range of bioactivities including antioxidative, anticarcinogenic, anti-inflammatory properties, which are mostly related to the potential health-promoting benefits against human health risks such as hypertension, obesity, cardiovascular diseases, diabetes, and cancer [12,13,14]. Heim et al. reported that the aglycones of flavonoids have a higher antioxidative activity than their glycosides [1]. Aglycones in the intestine can be metabolized by the human gut microbiota into different more simplified metabolites and thus can be effectively absorbed [15,16]. Both microorganisms used in fermentation media and catalytic reactions (e.g., glycosylation, deglycosylation, ring cleavage, methylation, glucuronidation, and sulfate conjugation) affect the generation of new byproducts converted from flavonoids [17,18,19]. Therefore, employing bacterial or fungal fermentation processes may only enhance the release of bound phenolic compounds from the plant cell walls but also help the conversion of phenolic compounds into different metabolites, which can exert other bioactivities. This contributes to the production of extracts and food products with a highly-added value by a controllable fermentation process with a pure microbial strain [20].

*Eurotium* sp., which has been commonly known as “golden flower”, is the main probiotic fungus traditionally used in preparing Fu brick dark tea. It is characterized by its xerophilic growth on a substrate at extremely low free water content. *Eurotium* sp. is a nontoxic and safe fungus, and can secrete many active metabolites, which strongly affect tea quality and offer many functional benefits for human health [21,22,23,24].

**Figure 2:**
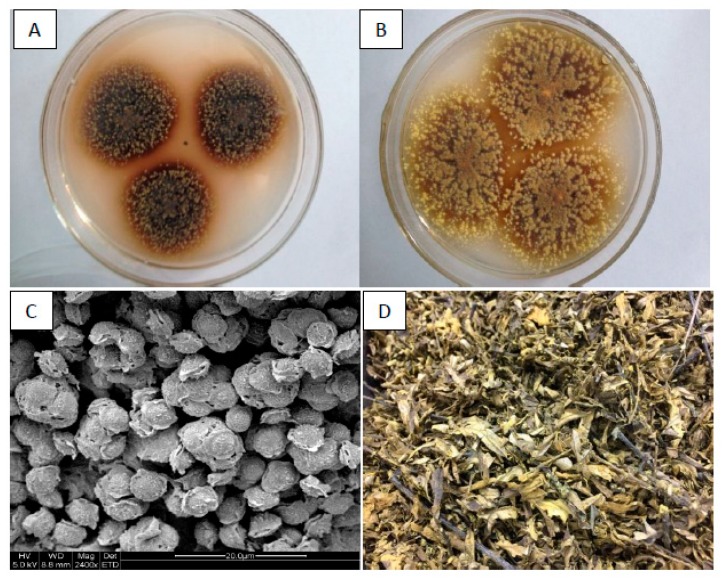
Morphological characteristics of *Eurotium amstelodami* BSX001 isolated from Fu brick tea. (**A**) Colonies grown on the CZA agar at 28 °C for ten days. (**B**) Colonies grown on the CZA20 agar at 28 °C for ten days. (**C**) SEM image of gold-sputtered samples of ascospores (incubated for two weeks), scale bar = 20 µm. (**D**) *Hippophae rhamnoides* leaves (HRL) with “golden flowers” visible to the naked eye.

Although *Eurotium* sp. has been proved to improve the bioactivities of many natural materials [25,26,27], few studies shed light on the metabolic modifications of compounds in the matrix by microorganisms. Thus, the objective of the present study was to elucidate the enhanced antioxidant capacity of HRL fermented by *E. amstelodami*. We first isolated several strains of *Eurotium sp.* from Fu brick dark tea. We then screened a novel strain *E. amstelodami* BSX001 with high glycosidase activity and employed it to ferment HRL (Figure 2). Furthermore, to understand the bioconversion pathway of flavone glycosides, analyses of relevant biochemical reactions catalyzed by *E. amstelodami* were performed using the standard compounds.

## 2. Materials and Methods

### 2.1. Sampling Site, Strain and Mediam

*Hippophae rhamnoides* leaves in September were collected from Zhangyi Town (E 106°28′, N 36°01′), Yuanzhou District, Guyuan City, Ningxia Hui Autonomous Region, China. The local altitude is above 2000 m and *H. rhamnoides* is widely distributed in this natural environment.

*Eurotium amstelodami* BSX001 was isolated from the Fu brick tea (Hunan Anhua Tea Factory, Changsha, China). The strain was cultured at 28 ℃ on the potato-dextrose agar (PDA) for seven days. The spores of *E. amstelodami* BSX001 were collected and stored as inoculums for subsequent use.

The selective medium used in this study was composed of: 0.1% geniposide, 1% sodium glutamate, 0.3% NaNO_3_, 0.1% K_2_HPO_4_, 0.05% KCl, 0.001% FeSO_4_ and 0.05% MgSO_4_ with geniposide as sole carbon source to screen the strain with high β-glucosidase activity. When geniposide is enzymatically hydrolyzed by β-glucosidase, genipin is produced by removing the glycosyl group at the end of the molecule. Genipin reacts with sodium glutamate in a series of rearrangements to produce gardenia blue pigment. The different fungi isolated from Fu brick tea were planted on the screening plate of geniposide and cultured at 28 °C for 5–10 days.

### 2.2. Preparation of E. amstelodami-Fermented HRL

*E. amstelodami* BSX001 was directly cultured on PDA plates in a 28 °C incubator for seven days. The golden mycelia were washed off from the plate with 15 mL sterilized water and the solution was transferred to a 50-mL sterile centrifuge tube. Then, the solution was filtered with absorbent cotton and adjusted to 1 × 10^7^ spores/mL [28]. HRL were put into flasks (40 g per flask) for autoclave sterilization at 121 °C for 20 min, thereafter, 3 mL of *E. amstelodami* spore solution were inoculated into each cooled flask. The flasks were placed at 28 °C to ferment for 7–30 days, thereafter, the fermented leaves were all dried at 80 °C for about 3 h until the water content was only 5%–7% (W/W) [28]. A control sample was prepared by inoculating with 3 mL of sterilized water and incubating at 28 ℃ for seven days. The experiment was carried out in triplicate.

### 2.3. Measurements of the Total Phenolic Compounds and Flavonoids

The content of total phenolic compounds was determined according to the method by Quettier-Deleu et al. [29]. Absorbance at 760 nm was tested by using the UV-3802 UV/Vis spectrophotometer (Uico Shanghai Instrument Co. Ltd., Shanghai, China). The content of total phenolic compounds was calculated as mg of gallic acid equivalent (GAE) on the basis of dry weight (DW) (mg GAE/g DW) from the calibration curve of the standard gallic acid.

The content of total flavonoids was determined with the method by Juan et al. [30]. A mixture of 1 mL of the extract (1 mg/mL), 2.0 mL of nano-pure water and 0.15 mL of 5% NaNO_2_ solution was prepared and allowed to react for 6 min at room temperature. Then, 0.15 mL of 10% AlCl_3_ solution was added and mixed thoroughly. After 6 min, 2 mL of 4% NaOH (w/v) solution was added and allowed to stand for another 15 min. Absorbance of the mixture was measured at 510 nm. The total content of flavonoids was calculated as milligrams of rutin equivalent (RTE) based on dry weight (mg RTE/g DW) from the calibration curve of the standard rutin.

### 2.4. Determination of Antioxidant Activities

Samples were all diluted by MeOH to 1 mg/mL, and all the tests carried out in the sterile 96-well plate. Each sample was tested three times with a microplate spectrophotometer (Multiskan FC, Thermo Scientific, Waltham, MA, USA).

The activity of radical scavenging was determined using 2, 2-diphenyl-1-picryl-hydrazyl-hydrate (DPPH) free radical method [31], with some modifications. Twenty micro-liters of extract were mixed with 1980 μL of methanolic solution of DPPH (75 μM). Decolorization of purple free radical DPPH solution was measured at 517 nm after 30 min incubation at room temperature in the dark. A Trolox calibration curve was done from 0.1 to 1 mg/mL. Results were calculated as mg of Trolox equivalents/g of dry extract (mg TE/g).

Assay of 2, 2’-azino-bis (3-ethylbenzothiazoline-6-sulphonic acid) (ABTS) was performed by the method by Re et al. [32]. Firstly, 7 mM ABTS diammonium salt and 2.4 mM potassium sulfate were mixed with an equal volume and allowed to stand in the dark for 12 h at room temperature to generate the fresh ABTS. One milliliter of fresh ABTS solution was mixed with 60 mL of methanol to obtain an absorbance of 1.10 ± 0.02 at 734 nm. Then, 150 μL of the extract (0.1 g/L) were allowed to react with 2850 μL of ABTS solution and the absorbance was measured at 734 nm after 2 h. The standard curve was linear when the concentration of Trolox ranged from 25 to 150 mg/L. Results were calculated by mg of Trolox equivalents/g of dry leaf.

The ferric reducing/antioxidant power (FRAP) assay was carried out by the method of Benzie and Strain [33], with some modifications. The stock solution was made by mixing the solution of 300 mM acetate buffer (pH 3.6) and 10 mM TPTZ (2, 4, 6-tripyridyl-s-triazine) with the solution of 40 mM HCl and 20 mM FeCl_3_.6H_2_O. The solution was preheated to 37 °C before use. Then, 150 μL of the extract (0.1 g/L) were allowed to react with 2850 μL of the FRAP reaction solution for 30 min in the dark. Absorbance of the colored product (ferrous tripyridyltriazine complex) was measured at 593 nm. The standard curve was linear when the concentration of Trolox ranged from 25 to 150 mg/L. Results were calculated by mg of Trolox equivalents/g of dry leaf.

### 2.5. Quantitative HPLC Analysis of Flavonoid Aglycones 

An Agilent 1260 series HPLC system (Agilent Technologies, Waldbronn, Germany) coupled with a diode array detector (DAD) and Vydac C18 column was used for flavonoid aglycones analysis. The binary gradient consisted of 0.2% phosphoric acid/water as solvent A and methanol as solvent B at a flow rate of 0.8 mL/min, following the elution program: 0–5 min (15% B), 5–15 min (15–30% B), 15–20 min (30% B) and 20–30 min (decreased from 30% to 15% B). The temperature of the column was maintained at 35 ℃. The injection volume was 10 μL for each solution. The chromatogram with retention time was recorded at 370 nm. Identification of compounds was referred to the retention time, co-injections, and spectral matching with standards. Standard stock solutions of compounds were prepared in methanol, filtered through 0.22 μL filters (Millipore), and appropriately diluted (0.01–100 μg/mL) to obtain the desired concentrations in the quantification range. The calibration graphs were plotted after linear regression of the peak areas versus concentrations.

### 2.6. Analysis of Bioconservation Products

To determine the bioconversion pathway of flavonoids, the standard compounds were used for fermentation by *E. amstelodami* in the Czapek–Dox medium containing the following standard compounds (rutin, quercetin 3-glucoside, quercetin, isorhamnetin-3-glucoside, isorhamnetin, kaempferol-3-glucoside, and kaempferol) [34]. Briefly, 0.5 mL of fresh *E. amstelodami* spore suspension was inoculated into 50 mL of Czapek–Dox medium and incubated at 30 °C for two days at 180 rpm. A volume of 0.25 mL methanol solution containing 8 mg of standard compounds was then supplemented into the medium and maintained for additional four days. After that, the culture was adjusted to 20 mL with fresh sugar-free Czapek–Dox medium and extracted twice with 10 mL of ethylacetate. The extract was immediately stored at 4 ℃ for further analysis. In addition, fermentation in the Czapek–Dox medium without the standard compound was treated as the control group.

To obtain pure products for chemical analysis, the fermentation experiment was performed in a 2000-mL flask. Rutin (2 g) and distilled water (200 mL) were separately added to each of six 2000-mL flasks. After fermentation, the flavonoid compounds were extracted with 60% ethanol. After filtration, the extract was concentrated in a rotary vacuum evaporator at 50 °C. Finally, methanol was used to re-dissolve the product. A Waters1525 series HPLC system (Waters, Milford, MA, USA) coupled with a photo-diode array detector (PDA) and XBridge C18 column was used for separation and collection the metabolites of rutin. Fifty percent methanol was used as mobile phase for equivalent elution. The temperature of the column was maintained at 30 °C. The injection volume was 500 μL. The chromatogram with retention time was recorded at 370 nm. Six reference compounds were isolated by semi-preparative HPLC. Each of the collected six flavonoids was divided into two sets: one used for analysis by NMR and MS to identify their chemical structures and the other used as a substrate for further metabolic analysis.

### 2.7. Structure Elucidation

^13^C and ^1^H NMR spectra were tested with a Bruker AC 400 Instrument at 30 °C using 3-mL tubes. Samples were dissolved in methanol-D4. The chemical shifts were calculated in parts per million (ppm) relative to tetramethyl silane (TMS). The MS instrument with a TOF analyzer was fitted to an electronic spray ionic (ESI) source and operated in negative ion mode with a capillary voltage at 3500 V and a skimmer voltage at 20 V. The nebulizer pressure was 40 psi and the nitrogen flow rate was 9 L/min. The temperature of drying gas was 400 °C. The monitored mass range was from m/z 70 to 1200.

### 2.8. Statistical Analysis

All tests of antioxidant activity were repeated in triplicate, and the data were expressed as the means ± SD (standard deviations). One way analysis of variance (ANOVA) and significant difference tests were performed using SPSS 22.0 software (SPSS Inc., Chicago, IL, USA). Significant differences were determined by an independent-sample *t*-test (*p* = 0.05) or Duncan’s multiple-range tests at *p* = 0.05. Mean differences were considered significant when *p* < 0.05. Principal component analysis (PCA) was applied to the mean values of the relative peak area ratios of seven flavonoids, and the results were analyzed by Origin 8.0 (Origin Lab, Hampton, MA, USA).

## 3. Results and Discussion

The leaves of *H. rhamnoides* (HRL) are rich in flavonoids, but most of them exist in the form of glycosides. Flavonoid aglycones can be obtained from HRL by enzymatic treatment or biotransformation. In this study, a screening method for plate coloring was designed to obtain a β-glucosidase-producing strain from fermented tea. The content of total flavonoids and phenolic compounds in HRL were increased after fermentation. In addition, flavonoid aglycones were released, and the antioxidant activity was significantly enhanced. In order to further clarify the modification of flavonoids by *E. amstelodami*, metabolites were prepared with rutin as substrate. We finally predicted its bioconversion pathway through the corresponding metabolic reactions.

### 3.1. Screening of β-Glucosidase-Producing Fungi

**Figure 3:**
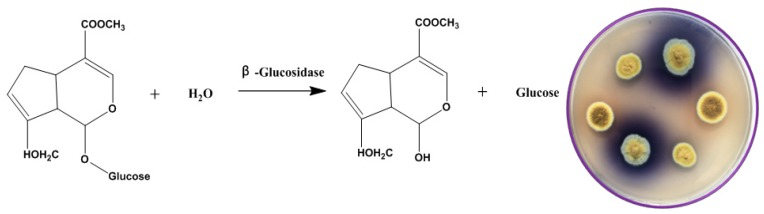
Biotransformation of geniposide into genipin by *Eurotium* sp. The plate on the right shows the colony morphology of *E. amstelodami* BSX001 on the selective medium with gardenoside as sole carbon source. Colonies with a larger blue circle had a higher glycosidase activity.

In nature, there are many fungi that can produce β-glucosidase to degrade geniposide. However, such microbial resources can be well exploited when an appropriate medium is designed or employed [35,36]. Using geniposide as a sole carbon source in the modified Czapek–Dox medium, we managed to screen a wild-type fungus that produces β-glucosidase to catalyze the hydrolysis reaction of geniposide to genipin (Figure 3). After the hydrolysis of geniposide by β-glucosidase, the generated genipin reacted with glutamate to form a blue transparent circle (Figure 3). This strain was identified as *E. amstelodami* BSX001 and preserved by the Agricultural Culture Collection of China (ACCC) under a collection number ACCC32729.

As flavonoids in plants usually exist as β-glycosides and most pretreatment procedures cannot lead to cleavage of the glycosidic linkage, flavonoids in foods are generally present as glycosides. Therefore, microorganisms that are capable of producing β-glucosidase play important roles in food and fermentation processes of flavonoids. For example, during the grade wine fermentation, microbial β-glucosidase was found to hydrolyze the glycosidic bond between terpenes and glucose, releasing terpenes to make the wine more fragrant [37]. Furthermore, microbial β-glucosidase can catalyze the release of isoflavone aglycones (e.g., genistein and daidzein) from the fermented soy-based products, especially tempeh [38]. In addition, β-glucosidase can break down the side glycosidic linkage of ginsenoside to enhance its anticancer activity, which has been found to be highly associated with the number of glycosyl [39]. Therefore, the development and application of β-glucosidase-producing microbes have a great significance in the food and fermentation industries.

### 3.2. Fermentation of HRL by E. amstelodami

**Figure 4:**
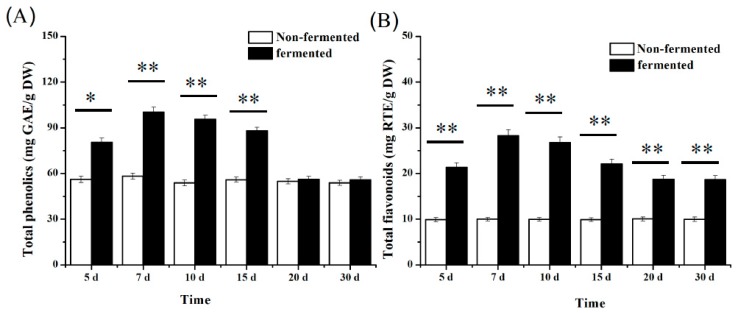
Contents of the total phenolic compounds (**A**) and flavonoids (**B**) in non-fermented and fermented HRLs by *E. amstelodami* BSX001. Note: ** extremely significant difference (*p* < 0.01) and * significant difference (*p* < 0.05).

The unique “flowering” process of Fu brick tea endows black tea with unique aroma and efficacy. This is closely related to the metabolism and transformation of some products, such as phenolic compounds and flavonoids, by *Eurotium* sp. Therefore, we tested the fermentation of HRL by *E. amstelodami* BSX001 and found that it grew well when using HRL as substrate (Figure 1). As shown in Figure 4A, the total content of phenolic compounds in the fermented HRL was significantly higher than that in the non-fermented HRL after seven days (*p* < 0.01). The total content of phenolic compounds in the non-fermented HRL was determined to be 55.97 ± 1.72 mg GAE/g DW within 30 days, whereas the total content in the fermented HRL peaked with 100.16 ± 3.25 mg GAE/g DW at seven days. The increase in the total content of phenolic compounds might be attributed to enhanced release of bounded phenolic compounds caused by hydrolytic enzymes produced by *E. amstelodami* BSX001 during fermentation [40,41]. However, the total content of phenolic compounds in the fermented HRL decreased after seven days and maintained at the same level as that in the non-fermented HRL after 20 days. This decrease would be caused by the catabolism of phenolic compounds by *E. amstelodami* BSX001.

Interestingly, the total content of flavonoids was observed to show similar patterns during the whole fermentation process (Figure 4B). The total content of flavonoids increased significantly after seven-day fermentation (*p* < 0.01), compared with that in the non-fermented HRL. Although the total content of flavonoids in the fermented HRL peaked at seven days, it also reduced thereafter and maintained at 20.18 ± 1.29 mg RTE/g DW, which was two folds of that in the non-fermented HRL. The decrease of the total content of flavonoids may be attributed to the oxidation of phenolic compounds catalyzed by the polyphenol oxidase in *E. amstelodami* [42]. Altogether, we suggest that the optimal fermentation period for HRL by *E. amstelodami* BSX001 should be no more than seven days.

### 3.3. Comparison of Antioxidative Activities

**Figure 5:**
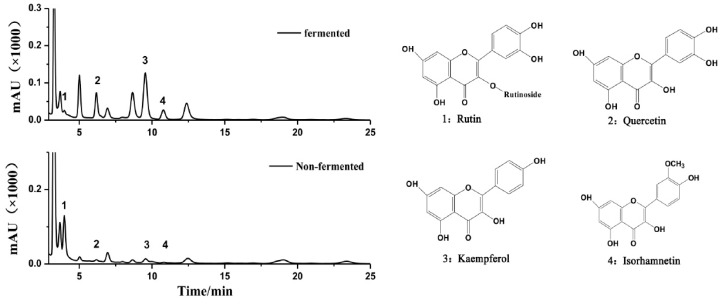
HPLC profiles of flavonol aglycones in non-fermented and fermented HRL. Numbers above the peak were corresponding to the compounds listed on the right.

Antioxidative activity is one of the most important factors to evaluate the quality of fermented tea. However, this activity typically depends upon the composition of chemical compounds in tea. After 7-day fermentation, the contents of some compounds in HRL increased, whereas those of other compounds increased, with some new products being generated (Figure 5). Among them, the content of rutin reduced from 4.61 ± 0.13 mg/g to 0.92 ± 0.05 mg/g after fermentation. The contents of quercetin, kaempferol, and isorhamnetin were detected to be 0.64 ± 0.01 mg, 0.48 ± 0.01 mg and 0.85 ± 0.03 mg, respectively, in one gram of dry fermented HRL, whereas the content of these flavonol aglycones in the non-fermented HRL was very low (Table 1). Furthermore, the three different evaluation standards showed that antioxidant activities of fermented HRL were enhanced significantly (*p* < 0.01, Table 1) compared with that of the non-fermented HRL. These findings were consistent with the report when *Angelica dahurica* was employed as the substrate [43], where it claimed that the increased content of phenolic compounds in the fermented samples would be responsible for the enhancement of antioxidant activities. Therefore, the changes in the composition of the fermented products by *E. amstelodami* BSX001 would greatly affect their bioactive properties.

Significant increase of flavonoid aglycones (e.g., quercetin, kaempferol and isorhamnetin, Supplementary Table S1) in the fermented HRL might have resulted from the deglycosylation of β-glucosidase released by *E. amstelodami* BSX001. As discussed above, most flavonoids in the non-fermented HRL are present in the form of flavonoid glycoside, which has been reported to inhibit free-radical mediated events, especially antioxidative activity. As these compounds differ both in the flavan nucleus and in the number, position, and types of substitutions that influence radical scavenging and chelating activity, their antioxidative activities are governed by their chemical structures. Furthermore, the flavonoid derivatives that have a hydroxyl group substituted on the C-ring were found to show high antioxidant activity. However, these bio-converted quercetin, kaempferol, and isorhamnetin did have a hydroxyl group at the C-3 position of the C-ring after fermentation, which further supported that the deglycosylation of β-glucosidase occurred in this bioconversion process.

The enhancement of antioxidative activity would be caused by the increase of flavonoid aglycones. The spatial arrangement of substituents in such compounds is perhaps a greater determinant to antioxidative activity than the flavan backbone alone. Consistent with most polyphenolic antioxidants, both the configuration and the number of hydroxyl groups substantially influence several mechanisms of antioxidative activity [2,44,45]. Despite the disparity among methods to assess activity, there is broad agreement that hydroxyl groups endow flavonoids with substantial radical scavenging ability. The flavonoids without any hydroxyl group (flavone, flavanone, and 8-methoxyflavone) or with the free hydroxyls only at C-5 and/or at C-7 (e.g., 5-hydroxyflavone, 7-hydroxyflavone, and 5, 7-dihydroxyflavone) had no scavenging of free radicals. Instead, flavonols with a free hydroxyl in the C-3 position showed high ability to scavenge DPPH radicals. Flavonoids with a 3-OH and 3, 4-catechol are reported to have 10-fold antioxidative activity of ebselen [46]. The superiority of quercetin in inhibiting both metal and nonmetal-induced oxidative damage is partially ascribed to its free 3-OH substituent [47,48]. It has been reported that radicals formed by H removal from hydroxyls at C-3 and C-4′ may be involved in the antioxidant properties of quercetin [49]. Therefore, the results of the present experiments reveal that glycosides may undergo enzymatic hydrolysis, which resulted in the formation of active aglycones that increased the total antioxidative activity of products. In addition, some secondary metabolites of *E. amstelodami*, such as anthraquinones and benzaldehyde derivatives, could also enhance the antioxidant activity of the fermented HRL.

### 3.4. Prediction of Bioconversion Pathways

After fermentation, the contents of flavonoid aglycones (e.g., quercetin, kaempferol, and isorhamnetin) in HRL increased, whereas the contents of flavonoid glycosides decreased. Therefore, it is possible that flavonoid glycosides might be converted further into aglycones or other compounds by *E. amstelodami*. To confirm the bioconversion pathway, we used rutin as a substrate, which is one of the flavonoid glycosides in HRL. As a result, six types of flavonoids were isolated from the metabolites by semi-preparative HPLC.

Six purified flavonoids were identified by 1H, 13C NMR and MS spectroscopy. Their structures were further confirmed by the NMR spectra data (Supplementary Materials). Compounds F2 and F3 were the yellow powder and were determined as quercetin 3-*O*-glucoside and isorhamnetin-3-*O*-glucoside, respectively, by comparing its NMR data with those reported previously [50]. Compound F4 was identified as kaempferol-3-*O*-glucoside. The NMR information of F5, F6, and F7 was in accordance with the standard of quercetin, isorhamnetin, and kaempferol, respectively. Therefore, the HPLC-MS-NMR analysis showed that quercetin-3-*O*-glucoside was the major metabolite when rutin was used as a substrate for *E. amstelodami*. Meanwhile, kaempferol-3-glucoside, kaempferol, isorhamnetin-3-*O*-glucoside, and isorhamnetin were also found in the products after fermentation. However, when quercetin-3-glucoside, isorhamnetin-3-*O*-glucoside or kaempferol-3-*O*-glucoside was used as a substrate, quercetin, isorhamnetin, and kaempferol were produced by *E. amstelodami*, respectively. In addition, *E. amstelodami* showed a poor metabolic ability to use quercetin and kaempferol.

**Figure 6:**
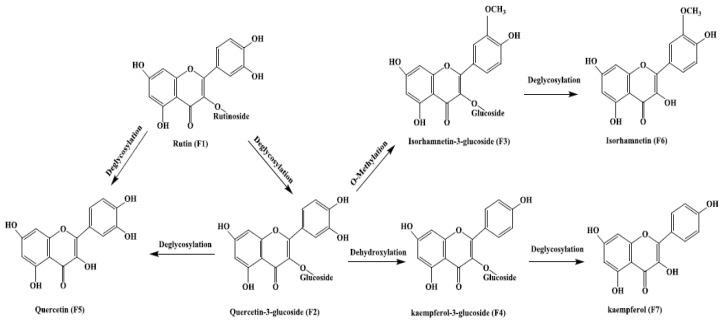
The proposed biotransformation pathway of rutin by *E. amstelodami*.

Based on the detected products, a biotransformation pathway of rutin in *E. amstelodami* was proposed (Figure 6). Rutin (F1) was first deglucosylated to yield quercetin-3-glucoside (F2), which was further modified by dehydroxylation and methylation to yield isorhamnetin-3-*O*-glucoside (F3) and kaempferol-3-*O*-glucoside (F4), respectively. Meanwhile, isorhamnetin-3-*O*-glucoside was subsequently deglycosylated to form isorhamnetin (F6) while kaempferol-3-glucoside was further transformed to kaempferol. Finally, quercetin (F5), isorhamnetin, and kaempferol (F7) were generated by deglycosylation. This metabolic pathway has greatly expanded the bioconversion of flavonoid glycosides in *E. amstelodami* and will provide further guidance for natural tea fermentation industry. In addition, exploration of the enzymatic properties of the *E. amstelodami* β-glucosidase and secondary metabolites with antioxidant activity would help elucidation of the enhancement of antioxidant activity.

## 4. Conclusions

In this study, a β-glucosidase-producing fungus, *Eurotium amstelodami* BSX001, was isolated from the Fu brick tea and was used to ferment HRL. The process of fermentation had greatly enhanced the antioxidant activity of final products. Furthermore, flavonoid aglycones (quercetin, kaempferol, and isorhamnetin) were significantly increased after fermentation. The bioconversion pathway revealed that rutin had been deglycosylated to form flavonoid aglycones by *E. amstelodami*. The fermentation of HRL by *E. amstelodami* can release more phenolic compounds and flavonoids, thereby improving the flavor and efficacy of fermented tea.

## Figures and Tables

**Table 1:** Contents of aglycones and antioxidant activities of non-fermented and fermented HRL.

HRL	Quercetin (mg/100 g dry leaf)	Kaempferol (mg/100 g dry leaf)	Isorhamnetin (mg/100 g dry leaf)	DPPH (mg Trolox equivalents/g dry leaf)	ABTS^+^ (mg Trolox equivalents/g dry leaf)	FRAP (mg Trolox equivalents/g dry leaf)
Fermented	64.14 ± 0.91	85.24 ± 1.62	48.37 ± 0.87	166.62 ± 3.60	188.32 ± 3.71	212.45 ± 4.15
Non-fermented	5.68 ± 0.01	11.02 ± 0.05	ND*	124.11 ± 2.15	135.67 ± 2.91	135.67 ± 2.91

Note: Data are presented as the mean ± standard deviation of three determinations. * ND indicated “not detected”.

## Supplementary Materials

The supplementary materials are available online at https://www.mdpi.com/2076-2607/7/5/122/s1.
