# Supplementary material for: Bioconversion of Flavonoid Glycosides from Hippophae rhamnoides Leaves into Flavonoid Aglycones by Eurotium amstelodami
Source: Microorganisms. 2019 May 5;7(5):122. doi: 10.3390/microorganisms7050122 (PMC6560391; doi:10.3390/microorganisms7050122)
Supplement: Supplementary file 1 [file microorganisms-07-00122-s001.pdf]

microorganisms

MDPI

Supplementary Materials

1. Identification of metabolites (F2, F3 and F4)

Compound F2 was the yellow powder with a molecular ion [M–H]– at m/z 463.The structure of F2 was further confirmed by the following NMR spectra data: ^{1}H NMR (400 MHz, MeOD) δ 7.71 (d, *J* = 2.1 Hz, 1H, 2′-H), 7.58 (dd, *J* = 8.5, 2.1 Hz, 1H, 6′-H), 6.86 (d, *J* = 8.5 Hz, 1H, 5′-H), 6.38 (d, *J* = 2.0 Hz, 1H, 8-H), 6.19 (d, *J* = 2.0 Hz, 1H, 6-H), 5.26 (d, *J* = 7.4 Hz, 1H, 1″-H), 3.72 – 3.23 (m, 6H, sugar protons).^{13}C NMR (101 MHz, MeOD) δ 179.46 (C-4), 165.98 (C-7), 163.00 (C-5), 158.99 (C-9), 158.43 (C-2), 149.83 (C-4′), 145.87 (C-3′), 135.61 (C-3), 123.19 (C-1′), 123.05 (C-6′), 117.56 (C-5′), 115.98 (C-2′), 105.66 (C-10), 104.31 (C-1″), 99.87 (C-6), 94.70 (C-8), 78.36 (C-5″), 78.09 (C-3″), 75.71 (C-2″), 71.19 (C-4″), 62.54 (C-6″). By a comparison of its NMR data with those reported previously, F2 was determined as quercetin 3-O-glucoside.

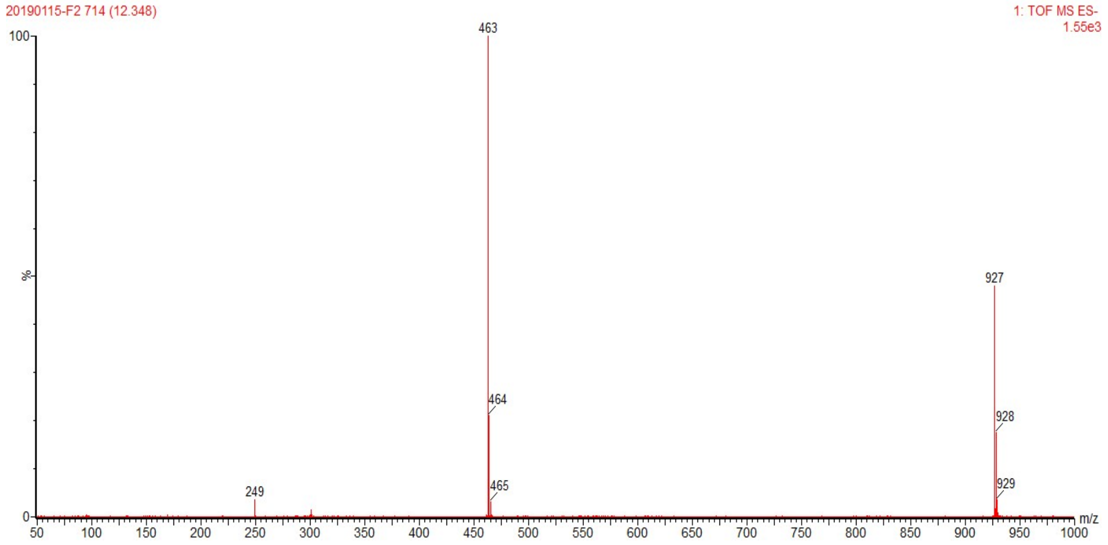

Figure S1. MS chromatograms of F2.

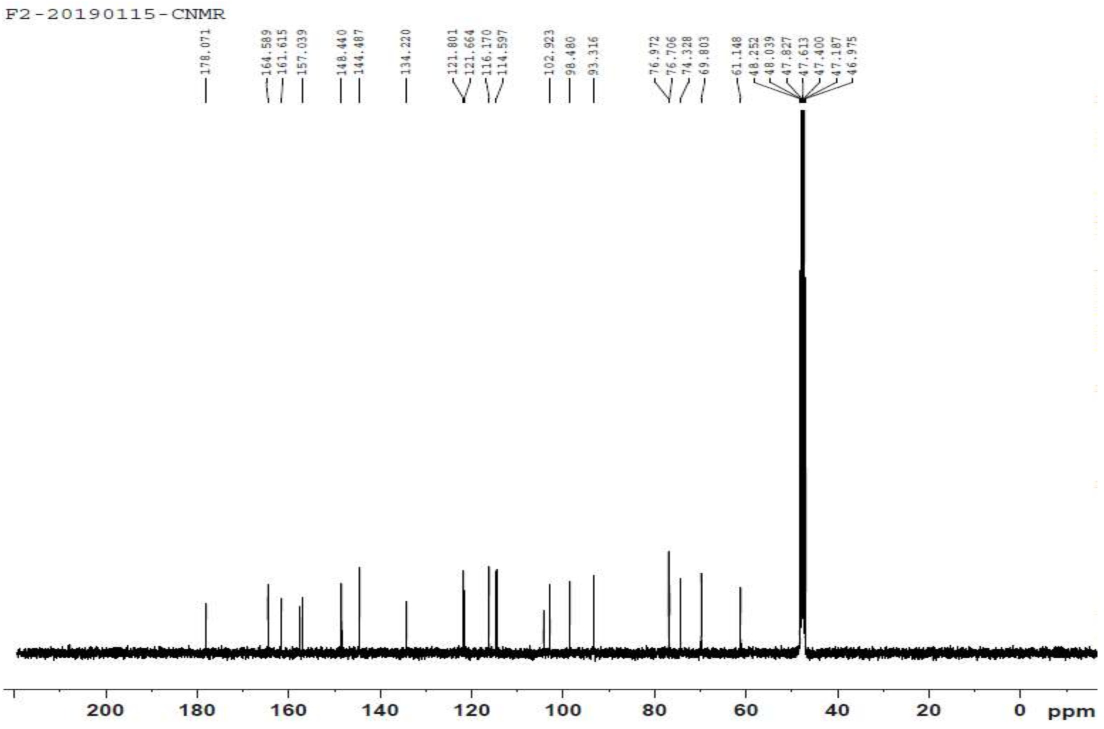

Figure S2. 13CNMR Spectra of F2.

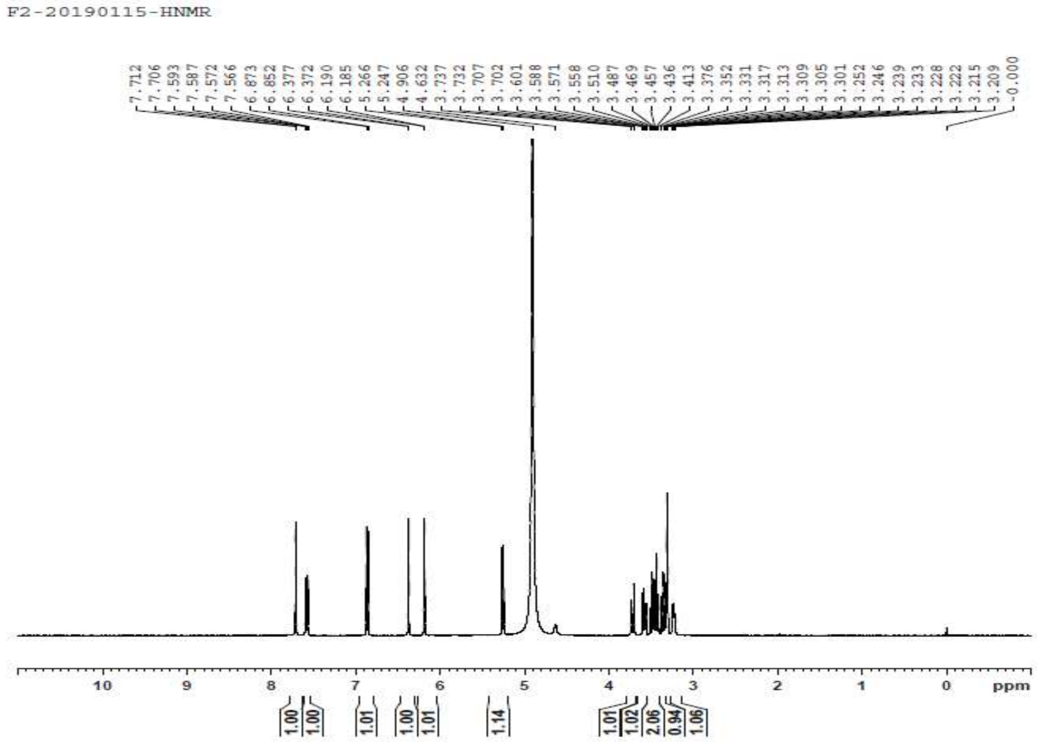

Figure S3. 1HNMR Spectra of F2.

Compound F3 was the yellow powder with a molecular ion [M–H]− at m/z 477. The data of NMR spectra were shown as follow:^{1}H NMR (400 MHz, MeOD) δ 7.93 (d, *J* = 2.0 Hz, 1H, 2′-H), 7.58 (dd, *J* = 8.5, 2.0 Hz, 1H, 6′-H), 6.90 (d, *J* = 8.5 Hz, 1H, 5′-H), 6.38 (d, *J* = 2.1 Hz, 1H, 8-H), 6.19 (d, *J* = 2.1 Hz, 1H, 6-H), 5.42 (d, *J* = 8.0 Hz, 1H, 1″-H), 3.94 (s, 3H, 4′-OCH_3), 3.74 – 3.25 (m, 6H, sugar protons).^{13}C NMR (101 MHz, MeOD) δ 179.42 (C-4), 165.95 (C-7), 163.08 (C-5), 158.63 (C-9), 158.44, 150.82 (C-4′), 148.37 (C-3′), 135.31 (C-3), 123.80 (C-6′), 123.08 (C-1′), 115.98 (C-5′), 114.36 (C-2′), 105.78 (C-10), 103.62 (C-1″), 99.85 (C-6), 94.72 (C-8), 78.54 (C-5″), 78.06 (C-3″), 75.92 (C-2″), 71.48 (C-4″), 62.54 (C-6″), 56.76 (4′-OCH_3). By a comparison of its NMR data with those reported previously, F2 was determined as isorhamnetin-3-O-glucoside.

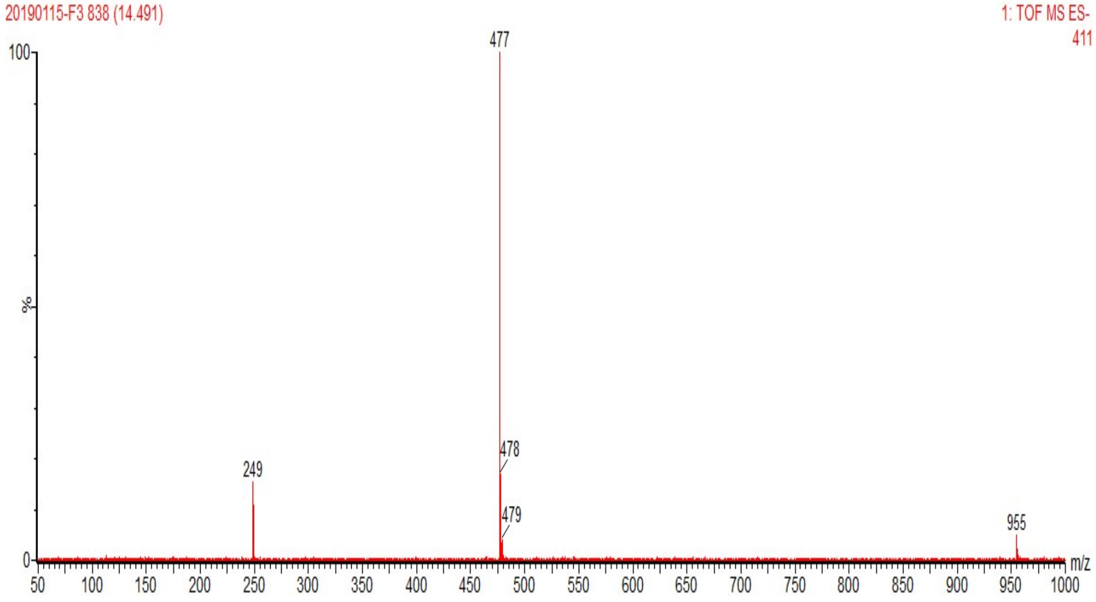

Figure S4. MS chromatograms of F3.

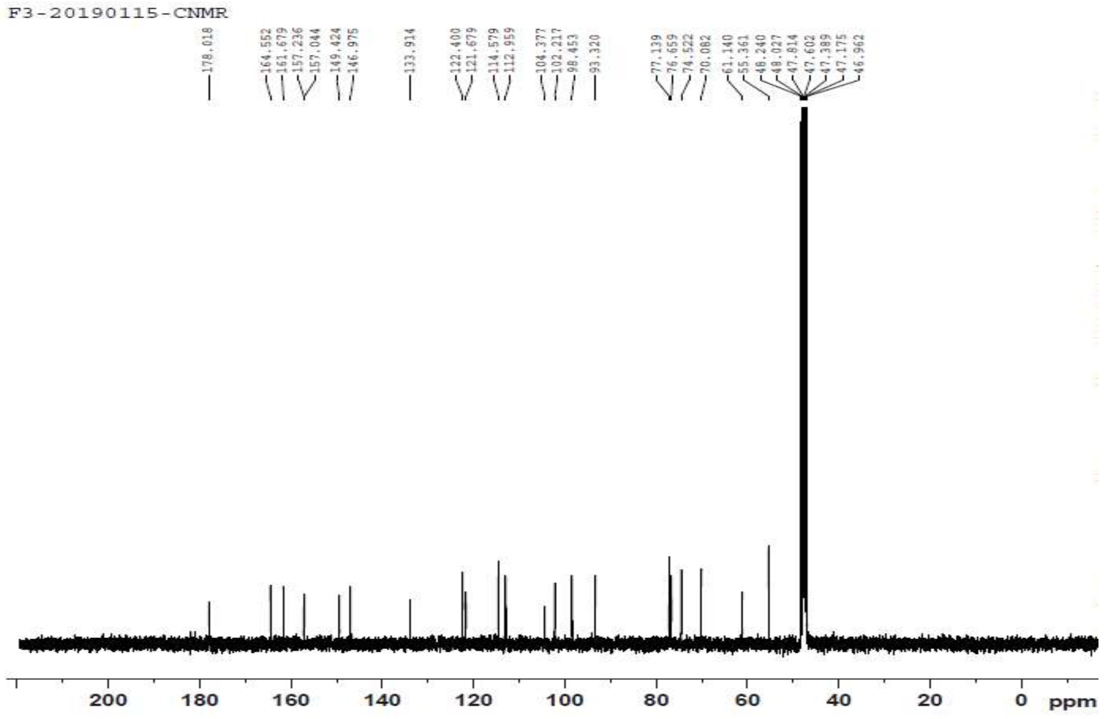

Figure S5. 13CNMR Spectra of F3.

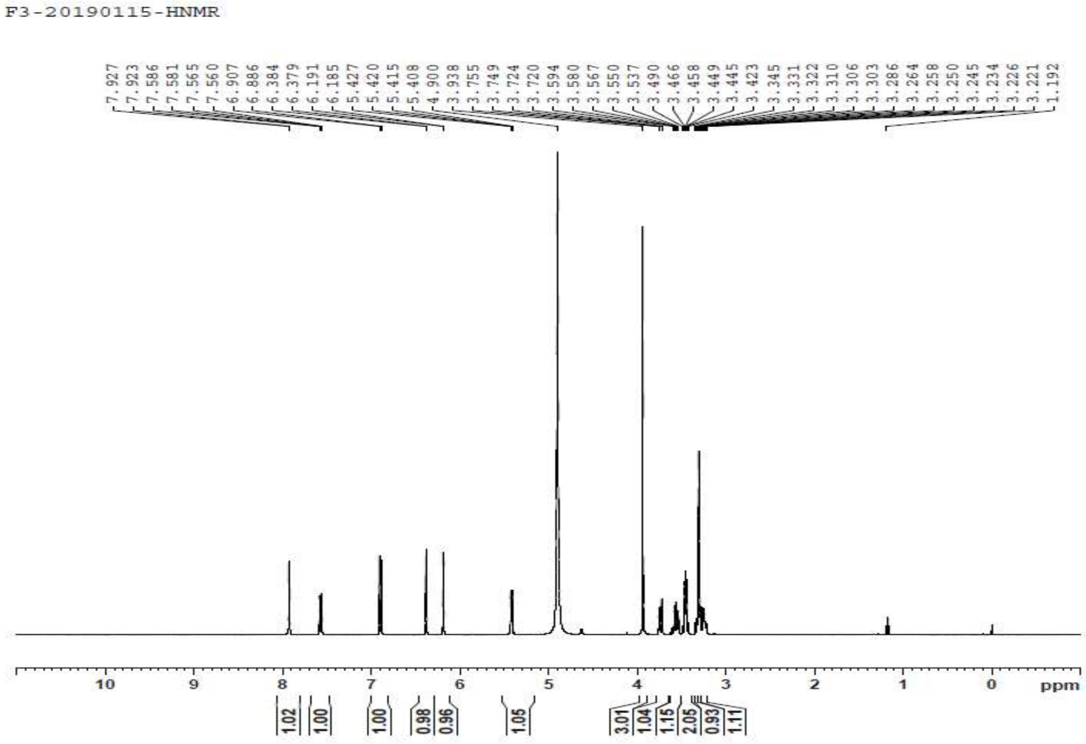

Figure S6. 1HNMR Spectra of F3.

The molecular weight of **Compound F4** was deduced to be 448 from the quasimolecular ion peak [M–H]− at m/z 447. The data of NMR spectra were shown as follow: ^{1}H NMR (400 MHz, MeOD) δ 8.06 (d, *J* = 8.9 Hz, 2H, 2′, 6′-H), 6.89 (d, *J* = 8.9 Hz, 2H, 3′, 5′-H), 6.41 (d, *J* = 2.1 Hz, 1H, 8-H), 6.21 (d, *J* = 2.1 Hz, 1H, 6-H), 5.27 (d, *J* = 7.3 Hz, 1H, 1′′-H), 3.70 – 3.21 (m, 6H, sugar protons).^{13}C NMR (101

MHz, MeOD) δ 179.55 (C-4), 166.06 (C-7), 163.11 (C-5), 161.58 (C-4′), 159.11 (C-9), 158.54 (C-2), 135.47 (C-3), 132.28 (2×C, C-2′, 6′), 122.82 (C-1′), 116.09 (2×C, C-3′, 5′), 105.75 (C-10), 104.07 (C-1″), 99.91 (C-6), 94.76 (C-8), 78.44 (C-5″), 78.05 (C-3″), 75.75 (C-2″), 71.38(C-4″), 62.64 (C-6″). Based on a comparison of its NMR data with previous report, compound F4 was identified as kaempferol-3-O-glucoside.

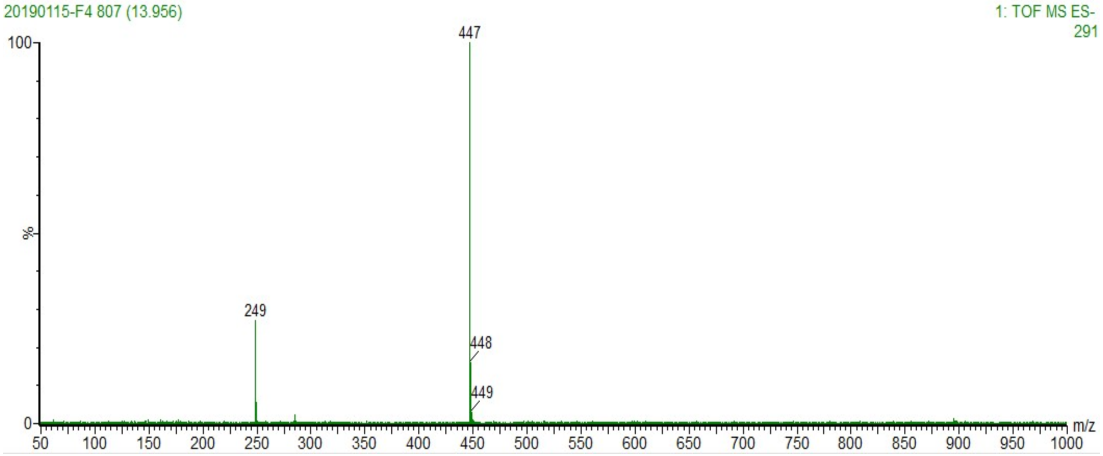

Figure S7. MS chromatograms of F4.

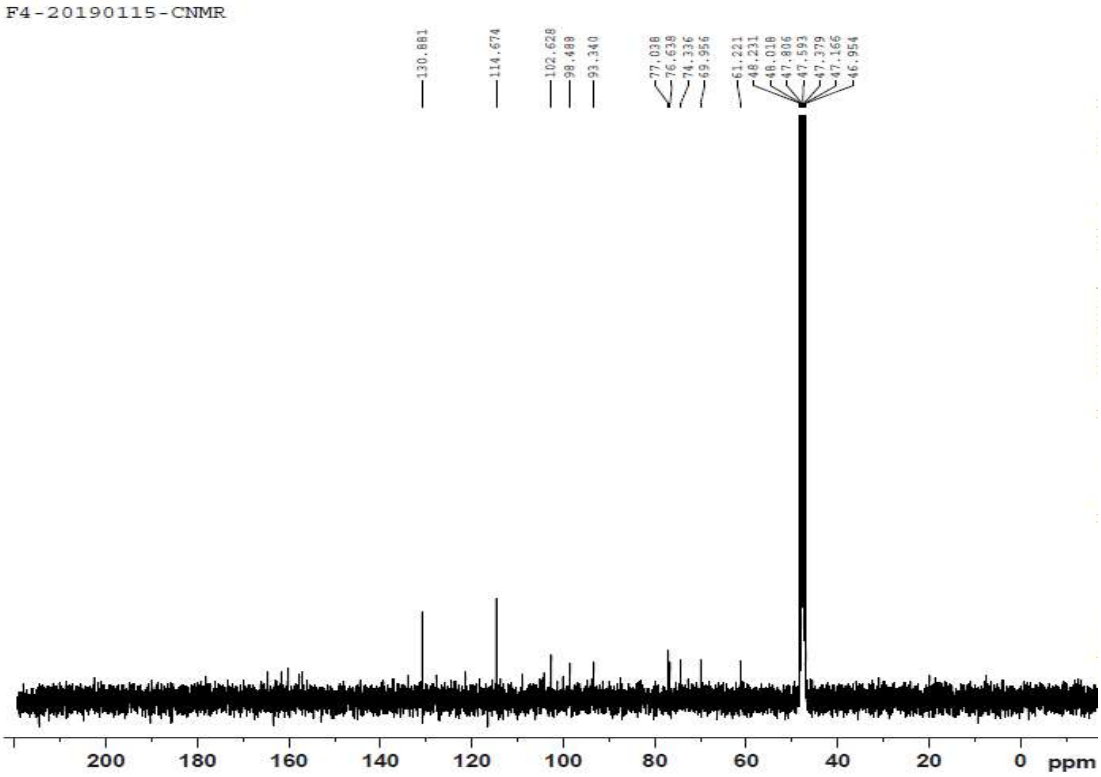

Figure S8. 13CNMR Spectra of F4.

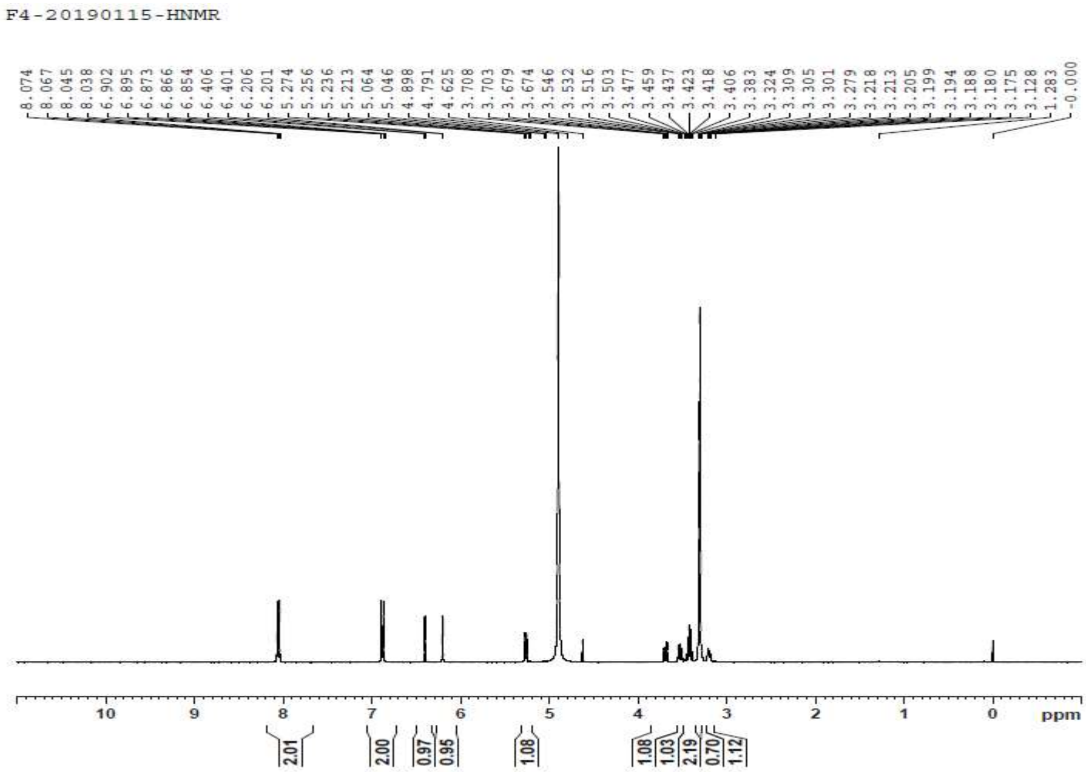

Figure S9. 1HNMR Spectra of F4.

2. Validation of calibration

2.1. Measurements of the total phenolics and flavonoids

Absorbance at 760 nm was test by using the UV-3802 UV/Vis Spectrophotometer (Uico Shanghai Instrument Co. Ltd., China). The content of total phenolics was calculated as mg of gallic acid equivalent (GAE) on the basis of dry weight (DW) (mg GAE/g DW) from the calibration curve of the standard gallic acid. Gallic acid showed good linearity in the range of 10-60 μg/mL, the regression equation was y=0.0108x+0.11181 (R^2=0.9978).

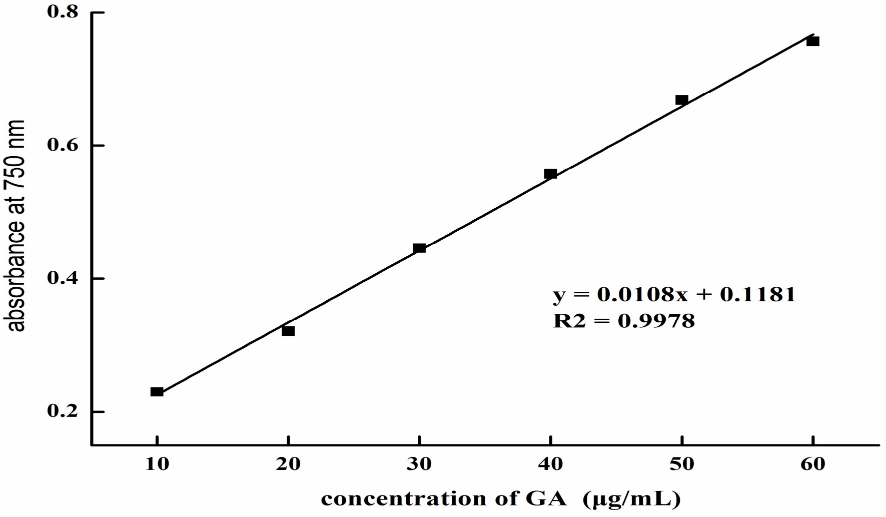

Figure S10. Standard curves of gallic acid.

Absorbance of the mixture was measured at 510 nm. Total content of flavonoids was calculated as milligrams of rutin equivalent (RTE) based on dry weight (mg RTE/g DW) from the calibration curve of the standard rutin. Rutin showed good linearity in the range of 20-200μg/mL, the regression equation was y=0.4007x+0.005 (R^2=0.9995).

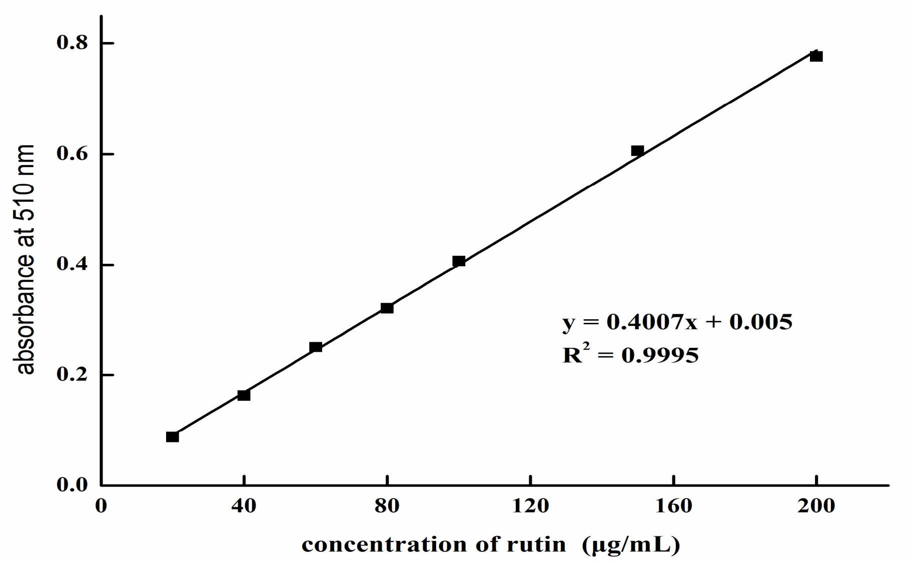

Figure S11. Standard curves of rutin.

2.2. Determination of antioxidant activities

2.2.1. DPPH assay

Decolourisation of purple free radical DPPH solution was measured at 517 nm. A trolox calibration curve was done from 0.1 to 1 mg/mL. Trolox showed good linearity in the range of 0.1-1 mg/mL, the regression equation was y=-0.3763x+0.4188 (R^2=0.9953).

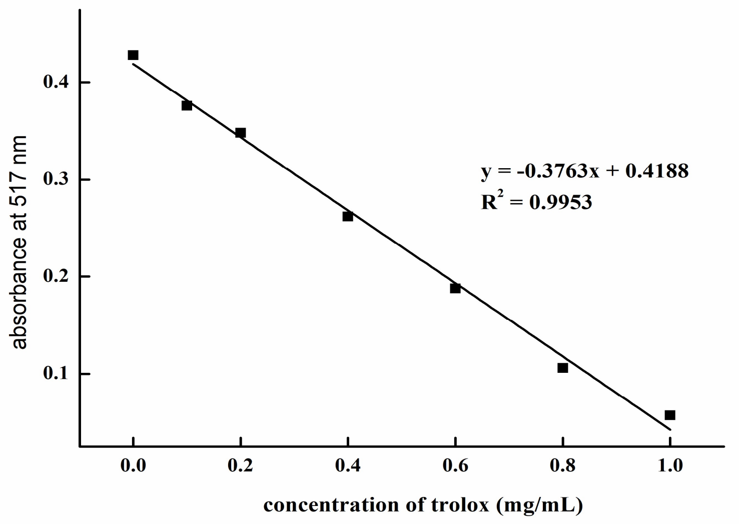

Figure S12. Standard curves of trolox.

2.2.2. ABTS assay

Decolourisation of free radical ABTS+ solution was measured at 734 nm. The standard curve was linear when the concentration of trolox ranged from 25 to 150 mg/L. The regression equation was y=-0.0052+0.8491 (R^2=0.9991).

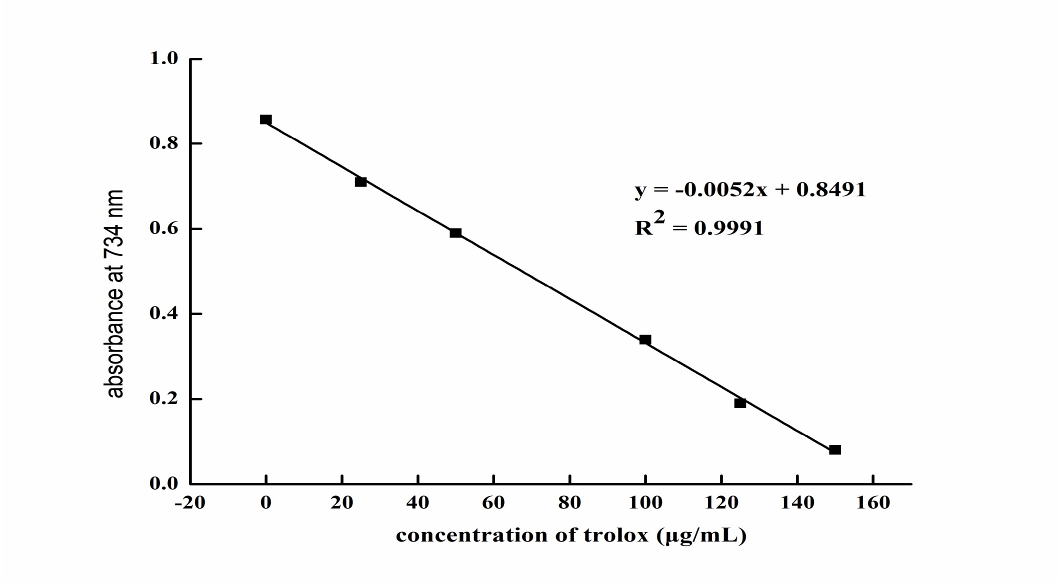

Figure S13. Standard curves of trolox.

2.2.3. FRAP assay

Absorbance of the colored product (ferrous tripyridyltriazine complex) was measured at 593 nm. The standard curve was linear when the concentration of trolox ranged from 25 to 150 mg/L. The regression equation was y=0.005x+0.1761 (R^2=0.9987).

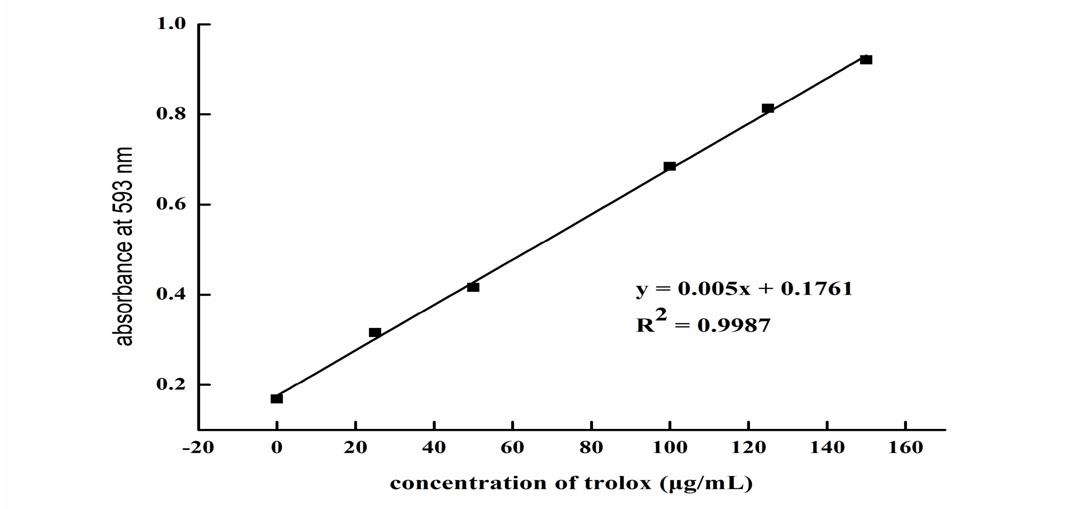

Figure S14. Standard curves of trolox.

2.3. Quantitative HPLC analysis of flavonoid aglycones

Quercetin showed good linearity in the range of 1.5-29.6 μg/mL, the regression equation was y=20.129x-3.4633 (R^2=0.9990), kaempferol showed good linearity in the range of 1.8-14.4 μg/mL, the regression equation was y=17.538x+0.554 (R^2=0.9997), isorhamnetin showed good linearity in the range of 2.4-19.1 μg/mL, and the regression equation was y=289.08x-76.883 (R^2=0.9998). The LODs of quercetin, kaempferol and isorhamnetin were 0.12 μg/mL, 0.15 μg/mL, 0.04 μg/mL. The LOQ of quercetin, kaempferol and isorhamnetin were 0.38 μg/mL, 0.94 μg/mL and 0.11 μg/mL, respectively.

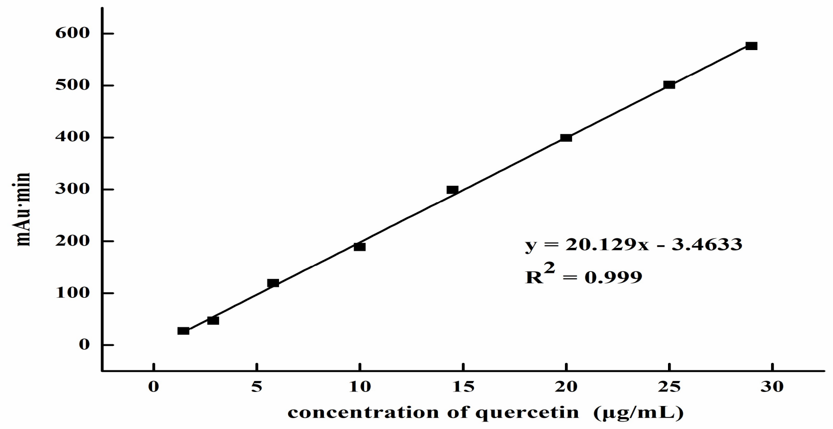

Figure S15. Standard curves of quercetin.

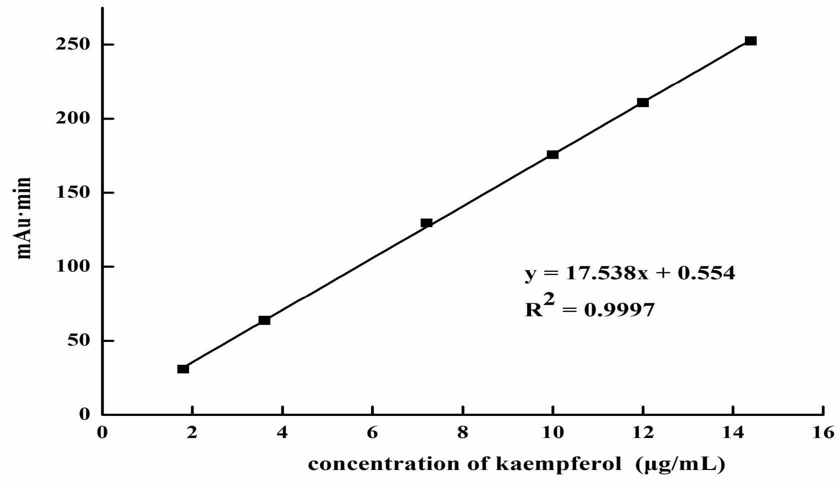

Figure S16. Standard curves of kaempferol.

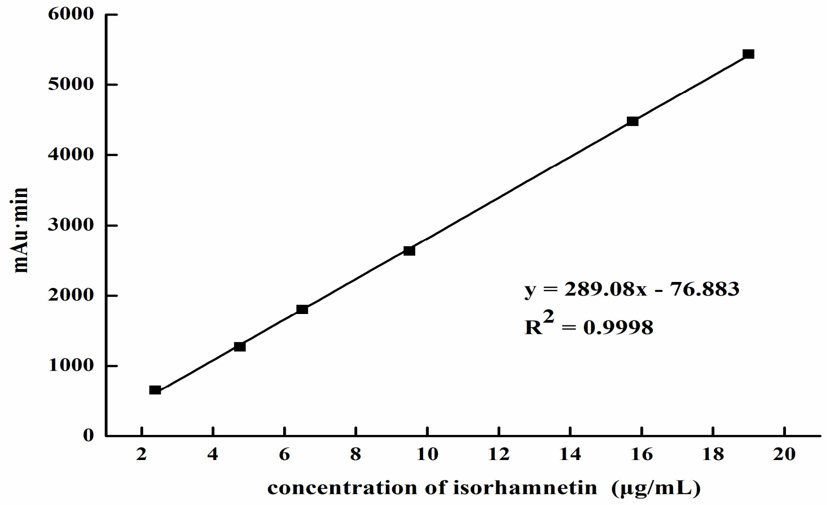

Figure S17. Standard curves of isorhamnetin.

2.4. Comparison of antioxidant activities between rutin and flavonoid aglycones

Table S1. Atioxidant activities of rutin and flavonoid aglycones.

**Flavonoids**	**DPPH (mg trolox equivalents/mg)**	**ABTS$^+$ (mg trolox equivalents/mg)**	**FRAP (mg trolox equivalents/mg)**
rutin	2.13±0.04	1.69±0.05	1.87±0.04
quercetin	5.18±0.11	2.95±0.14	3.24±0.12
kaempferol	2.69±0.09	1.98±0.06	2.15±0.08
isorhamnetin	2.51±0.05	2.11±0.09	2.07±0.05

© 2019 by the authors. Submitted for possible open access publication under the terms and conditions of the Creative Commons Attribution (CC BY) license (http://creativecommons.org/licenses/by/4.0/).
